# Baseline HbA1c is associated with glycemic response to digital personalized nutrition in type 2 diabetes: A real-world study

**DOI:** 10.1016/j.jtumed.2026.09.006

**Published:** 2026-09-22

**Authors:** Indira Kaibagarova, Aigul Sartayeva, Nurgul Ablakimova, Gaziza Smagulova, Alla Ovsiannikova

**Affiliations:** a Department of Pharmacology, Clinical Pharmacology, West Kazakhstan Marat Ospanov Medical University, Aktobe, Kazakhstan; b Department of General Medical Practice No. 2, West Kazakhstan Marat Ospanov Medical University, Aktobe, Kazakhstan; c The Institute of Internal and Preventive Medicine, Institute of Cytology and Genetics SB RAS, Novosibirsk, Russian Federation

**Keywords:** السكري من النوع الثاني, الصحة الرقمية, الأدلة من واقع الممارسة السريرية, HbA1c, الاستجابة للعلاج, تباين الاستجابة, Digital health, HbA1c, Heterogeneity, Real-world evidence, Treatment response, Type 2 diabetes

## Abstract

**Background and aims:**

Digital personalized nutrition is increasingly implemented in type 2 diabetes care, but the therapeutic effects appear to be heterogeneous in real-world settings. The aims of this study were to evaluate variations in the glycemic response to a digital personalized dietary intervention and identify baseline determinants of treatment effectiveness.

**Methods:**

In this prospective real-world before–after study, 47 adult outpatients with type 2 diabetes received individualized dietary recommendations generated by a web-based platform, with unchanged pharmacotherapy. Glycated hemoglobin (HbA1c), dietary intake, and body mass index were assessed at baseline and after 2–3 months. Subgroup analyses were performed according to baseline HbA1c (<7.0% vs ≥ 7.0%).

**Results:**

The intervention was associated with significant reductions in energy intake and body mass index. The overall change in HbA1c at the population level was modest, but a pronounced and statistically significant reduction was observed in patients with baseline HbA1c ≥ 7.0% (*p* = 0.003), whereas no meaningful change occurred in patients with well-controlled baseline glycemia. Baseline HbA1c was associated with the magnitude of the glycemic response to the intervention.

**Conclusions:**

Digital personalized nutrition in routine clinical practice resulted in heterogeneous glycemic effects rather than a uniform population-level response. Clinically meaningful improvement was primarily observed in patients with elevated baseline HbA1c, supporting a targeted implementation strategy and highlighting the importance of patient stratification in digital dietary interventions.

## الملخص

**أهداف البحث:**

هدفت هذه الدراسة إلى تقييم التباين في الاستجابة السكرية لتدخل غذائي رقمي مخصص لدى مرضى السكري من النوع الثاني، وتحديد العوامل الأساسية المرتبطة بمدى فعالية هذا التدخل، مع التركيز بصورة خاصة على مستوى السكر التراكمي (HbA1c) قبل بدء التدخل.

**طرق البحث:**

أُجريت دراسة مستقبلية من نوع قبل–بعد في ظروف الممارسة السريرية الواقعية، وشملت التحليلات النهائية 47 مريضاً بالغاً من مرضى السكري من النوع الثاني في العيادات الخارجية. تلقى المشاركون توصيات غذائية فردية أُعدت باستخدام منصة إلكترونية، مع إبقاء العلاج الدوائي المضاد للسكري دون تغيير. وتم قياس السكر التراكمي (HbA1c) والمدخول الغذائي ومؤشر كتلة الجسم عند خط الأساس وبعد نحو 2−3 أشهر. كما أُجري تحليل فرعي وفقاً لمستوى HbA1c الأساسي إلى مجموعتين أقل من 7.0%، و7.0% أو أكثر.

النتائجأدى التدخل إلى انخفاض ذي دلالة إحصائية في إجمالي مدخول الطاقة اليومي، من وسيط بلغ 3044.9 إلى 2755.2 كيلو كالوري/يوم (P < 0.001)، كما انخفض مؤشر كتلة الجسم من 33.0 إلى 32.0 كغ/م² (P = 0.005). في المقابل، لم يكن الانخفاض في معدل السكر التراكمي (HbA1c) لدى العينة ككل ذا دلالة إحصائية؛ إذ انخفض من 7.17% إلى 7.02% (P = 0.130).

أظهر التحليل وفق مستوى التحكم السكري التراكمي اختلافاً واضحاً في الاستجابة. فقد بلغ وسيط التغير في HbA1c نحو −0.42% لدى المرضى الذين كان HbA1c لديهم 7.0%≥، مقارنةً بـ +0.17% لدى من كان HbA1c لديهم 7.0%<، وكان الفرق بين المجموعتين ذا دلالة إحصائية (P = 0.003). كما تحققت استجابة سكرية ذات أهمية سريرية، عُرّفت بانخفاض HbA1c بمقدار ≥0.5%، لدى 15 من أصل 47 مريضاً (31.9%)؛ وبلغت النسبة 50.0% لدى المرضى ذوي HbA1c الأساسي ≥7.0% مقابل 5.3% فقط لدى المجموعة ذات HbA1c <7.0%.

**الاستنتاج:**

أظهر التدخل الغذائي الرقمي المخصص تأثيراً متفاوتاً على التحكم السكري، وليس استجابة موحدة لدى جميع المرضى. وارتبط ارتفاع مستوى السكر التراكمي باستجابة سكرية أكبر، مما يشير إلى أن المرضى ذوي التحكم السكري غير الأمثل قد يكونون الأكثر استفادة من هذا النوع من التدخلات. ومع ذلك، ونظراً للتصميم الرصدي قبل–بعد، وغياب مجموعة ضابطة، ومحدودية حجم العينة، ينبغي تفسير النتائج باعتبارها ارتباطات وليست دليلاً على علاقة سببية أو قدرة تنبؤية مستقلة، مع ضرورة تأكيدها في دراسات مستقبلية أكبر تتضمن مجموعات ضابطة مناسبة.

## Introduction

Type 2 diabetes mellitus (T2DM) remains a major contributor to cardiovascular morbidity and healthcare burden worldwide. Alongside pharmacological treatment, dietary behavior is a central determinant of glycemic control, body weight, and long-term cardiometabolic risk. Although evidence-based dietary guidelines for T2DM are well established, their implementation in routine outpatient practice is frequently limited by time constraints, insufficient personalization, and low long-term adherence.^1^, ^2^. As a result, lifestyle interventions in real-world settings often achieve modest and heterogeneous metabolic effects.^3^, ^4^, ^5^

In recent years, digital personalized nutrition platforms have emerged as scalable tools to support dietary behavior change through individualized recommendations and automated feedback.^6^, ^7^, ^8^ Such approaches are particularly attractive for routine outpatient care, where resources for intensive dietary counseling are limited. However, most studies evaluating digital dietary interventions in T2DM have primarily focused on average changes in glycated hemoglobin or body weight, while evidence from real-world settings suggests substantial inter-individual variability in metabolic response.^9^, ^10^ The mechanisms underlying this heterogeneity remain insufficiently understood.

Despite growing evidence supporting digital dietary interventions, their clinical usefulness in routine care remains uncertain, as treatment effects are not uniform across patients.^11^ Identifying baseline characteristics associated with metabolic response is therefore essential for translating digital nutrition into clinical decision-making and for targeting interventions to patients most likely to benefit.^12^

Therefore, the present real-world before–after study aimed to assess both the overall metabolic effects of digital personalized diet therapy and the heterogeneity of glycemic response according to baseline metabolic status. Specifically, we investigated whether changes in glycated hemoglobin depend on baseline glycemic control and whether they are associated with changes in total energy intake or body mass index under conditions of unchanged pharmacotherapy.^13^

## Materials and Methods

### Study design

This prospective real-world before–after study was designed to evaluate within-patient changes under routine clinical care conditions. The absence of a parallel control group reflected the pragmatic nature of the study and allowed the assessment of intervention effects in real-world practice without altering standard care pathways.

### Study population

Adult outpatients with type 2 diabetes mellitus and receiving routine ambulatory care were eligible for inclusion. Patients treated with insulin therapy were excluded. Participants were recruited consecutively in routine clinical practice. All participants were managed exclusively in an outpatient setting, and antidiabetic pharmacotherapy remained unchanged throughout the observation period, with no dose adjustments or treatment intensification during follow-up.

### Sample size

The sample size was estimated using G∗Power (version 3.1.9.4) based on a paired before–after comparison of glycated hemoglobin (HbA1c) as the primary outcome. The calculation assumed a moderate within-subject effect size (dz = 0.44), corresponding to an expected reduction in HbA1c of approximately 0.3–0.4%, with an assumed standard deviation of the paired differences of 0.7–0.8%, a two-sided alpha level of 0.05, and statistical power of 80%. Based on these assumptions, the minimum required sample size was 40 participants. To account for potential attrition and incomplete follow-up, 75 patients were initially enrolled. Complete paired baseline and follow-up data were available for 47 participants, who were included in the final analysis.

### Digital personalized diet intervention

Individualized dietary recommendations were prepared using the NutriLogic web-based nutrition platform. The platform records anthropometric data, estimates energy requirements, and analyzes the habitual dietary intake of patients based on a structured questionnaire covering food consumption frequency and portion size. Nutrient intake was calculated using the Skurikhin food composition tables supplemented with the USDA FoodData Central database. Based on these data, the platform generated a 30-day dietary plan specifying the daily energy intake and macronutrient distribution. Dietary assessment was performed at baseline and repeated at follow-up. Nutritional recommendations were revised when changes in dietary intake were identified.

### Data collection and study outcomes

Dietary intake was assessed at baseline and follow-up using the NutriLogic platform. Nutrient intake was estimated based on the self-reported food consumption recorded for patients through the platform. Anthropometric assessment included the body mass index (BMI), and glycemic control was evaluated using HbA1c.

Follow-up assessment was scheduled for approximately three months after baseline. The interval between visits was available for 45 of 47 participants and the average was 90.1 ± 22.3 days (median: 92 days, interquartile range (IQR): 84–100; range: 47–147 days).

The primary outcome was the change in HbA1c. Secondary outcomes included changes in total energy intake, dietary composition, and BMI. Prespecified subgroup analyses were performed according to baseline HbA1c (<7.0% vs ≥ 7.0%) to explore potential differences in treatment responses.

### Statistical analysis

Statistical analyses were performed using SPSS (version 26.0, IBM Corp., Armonk, NY, USA) and RStudio (R version 4.3.2). Due to the non-normal distributions of the variables, non-parametric methods were applied. Paired comparisons were conducted between baseline and follow-up measurements using the Wilcoxon signed-rank test. Associations between changes in dietary intake, anthropometric parameters, and HbA1c were evaluated using Spearman's rank correlation coefficients.

The primary analysis focused on within-patient changes in HbA1c. Responder analysis was also performed. A clinically meaningful glycemic response was defined as a reduction in HbA1c of at least 0.5% from baseline. The proportion of responders was calculated for the overall cohort and separately for participants with baseline HbA1c < 7.0% and ≥7.0%. Secondary analyses included changes in dietary intake, BMI, subgroup comparisons, and correlation analyses.

All statistical tests were two-sided, and a *p*-value <0.05 was considered to indicate a statistically significant difference.

### Ethical considerations

The study was conducted in accordance with the principles of the Declaration of Helsinki and approved by a local ethics committee (protocol No. 16-2024). All participants provided written informed consent prior to inclusion in the study.

## Results

### Baseline characteristics

The baseline demographic and clinical characteristics of the study population are summarized in Table 1. The median age of participants was 64 years, and women comprised 59.6% of the cohort. The median duration of type 2 diabetes mellitus was 2.0 years, reflecting a relatively early stage of the disease in routine outpatient care. At baseline, participants had obesity-level BMI values and variable glycemic control, with HbA1c values spanning both controlled and poorly controlled ranges. Among the 75 initially enrolled patients, 47 (62.7%) returned for the follow-up visit and were included in the final analysis, whereas the remaining 28 patients did not return and were excluded due to loss at follow-up. The baseline characteristics did not differ significantly between participants who completed follow-up and those who did not, including age, diabetes duration, BMI, and baseline HbA1c (all *p* > 0.05), suggesting no substantial attrition bias.

**Table 1:** Baseline demographic and clinical characteristics of the study population (N = 47).

Variable	Value
Age, years	64 (55–68)
Female sex, n (%)	28 (59.6)
Duration of diabetes, years	2.0 (0.5–5.0)
Body mass index, kg/m^2^	33.0 (28.36–36.60)
HbA1c, %	7.17 (6.66–7.89)

### Changes in energy intake, anthropometric parameters, and glycemic control

The changes in total energy intake, BMI, and HbA1c following digital personalized diet therapy are presented in Table 2. Overall, no statistically significant change in HbA1c was observed at the population level. By contrast, the total daily energy intake decreased significantly, and it was accompanied by a statistically significant reduction in BMI.

**Table 2:** Changes in energy intake, BMI, and HbA1c after digital personalized diet therapy (N = 47).

Variable	Baseline (median, IQR)	Follow-up (median, IQR)	Z	*p*
Energy, kcal/day	3044.9 (2581.3–3730.8)	2755.2 (2158.9–3180.8)	−5.09	<0.001
BMI, kg/m^2^	33.0 (28.36–36.60)	32.0 (27.00–36.00)	−2.79	0.005
HbA1c, %	7.17 (6.66–7.89)	7.02 (6.47–7.78)	−1.51	0.130

Data represent the median (interquartile range, IQR). Paired comparisons were performed using the Wilcoxon signed-rank test.

A clinically meaningful reduction in HbA1c (≥0.5%) was observed in 15 of 47 participants (31.9%). The proportion of responders differed markedly according to the baseline glycemic status, ranging from 5.3% (1/19) in participants with baseline HbA1c < 7.0%–50.0% (14/28) in those with baseline HbA1c ≥ 7.0%.

### Changes in macronutrient intake and dietary fat quality

Changes in dietary macronutrient intake were observed following the intervention (Table 3). Reductions were observed in protein, total fat, carbohydrates, sugars, and dietary fiber intake.

**Table 3:** Changes in macronutrient intake after digital personalized diet therapy.

Variable	Baseline (median, IQR)	Follow-up (median, IQR)	Z	*p*
Protein, g/day	124.49 (103.65–146.49)	105.36 (92.44–130.44)	−5.16	<0.0001
Total fat, g/day	165.33 (138.05–230.89)	152.29 (117.72–186.50)	−3.98	<0.0001
Carbohydrates, g/day	236.89 (211.11–287.45)	220.79 (165.79–249.26)	−4.49	<0.0001
Sugars, g/day	52.98 (43.40–61.65)	45.51 (34.03–52.89)	−4.04	0.0001
Dietary fiber, g/day	18.28 (16.40–22.36)	16.50 (12.54–19.46)	−4.10	<0.0001

Data represent the median (interquartile range, IQR). Paired comparisons were performed using the Wilcoxon signed-rank test.

The changes in dietary fat quality are presented in Table 4. Decreases in the intake of saturated, monounsaturated, and polyunsaturated fatty acids were observed during follow-up. All observed changes were statistically significant according to the Wilcoxon signed-rank test.

**Table 4:** Changes in dietary fat quality after digital personalized diet therapy.

Variable	Baseline (median, IQR)	Follow-up (median, IQR)	Z	*p*
Saturated fat, g/day	57.65 (48.41–85.41)	53.26 (39.50–64.22)	−4.38	<0.0001
Monounsaturated fat, g/day	34.29 (27.86–48.52)	31.15 (22.95–37.19)	−4.83	<0.0001
Polyunsaturated fat, g/day	44.41 (29.05–57.09)	43.71 (27.47–54.01)	−2.60	0.0086

Data represent the median (interquartile range, IQR). Paired comparisons were performed using the Wilcoxon signed-rank test.

### Subgroup and correlation analyses

Subgroup analysis according to baseline glycemic control indicated differences in the glycemic response to the intervention (Table 5). A significant reduction in HbA1c was observed exclusively in patients with baseline HbA1c ≥ 7.0%, whereas no meaningful change was detected among those with baseline HbA1c < 7.0%. The magnitude of the reduction in HbA1c was significantly greater in patients with baseline HbA1c ≥ 7.0% compared with those with better baseline control (see Table 6).

**Table 5:** Changes in outcomes according to baseline glycemic control.

Outcome	HbA1c *<* 7.0% (n = 19)	HbA1c ≥ 7.0% (n = 28)	Z	*p*
ΔEnergy, kcal/day	−362.7 (−576.7; 0.0)	*−*430.1 (*−*658.5; *−*119.4)	*−*0.87	0.385
ΔBMI, kg/m^2^	0.00 (*−*1.00; 0.30)	*−*0.53 (*−*1.98; *−*0.05)	*−*1.65	0.099
ΔHbA1c, %	0.17 (*−*0.21; 0.45)	*−*0.42 (*−*0.95; *−*0.01)	*−*3.01	0.003

Data represent the median (interquartile range). Between-group comparisons were performed using the Mann–Whitney U test.

**Table 6:** Additional analyses of glycemic response (effect size with 95% confidence interval (CI), responder analysis, and multivariable regression).

Group	n	Effect size (r)	*p*	95% CI
Total population	47	0.22	0.130	0.015–0.488
Baseline HbA1c < 7.0%	19	0.295	0.205	0.018–0.665
Baseline HbA1c ≥ 7.0%	28	0.480	0.0129	0.129–0.781

Effect size r was calculated as r = Z/√N (baseline HbA1c <7.0%: Z = 1.288, *p* = 0.205; baseline HbA1c ≥ 7.0%: Z = −2.539, *p* = 0.0129).

Changes in HbA1c were not significantly correlated with changes in total energy intake or BMI (all r < 0.2, *p* > 0.2), suggesting that the glycemic response was not directly driven by caloric reduction or weight loss.

Figure 1 illustrates the variable glycemic response to digital personalized diet therapy according to baseline glycemic control. Patients with baseline HbA1c ≥ 7.0% exhibited a consistent downward decrease in their HbA1c values, whereas no systematic change was found in patients with baseline HbA1c < 7.0%. These individual-level data highlight the substantial inter-individual variability.

**Figure 1:**
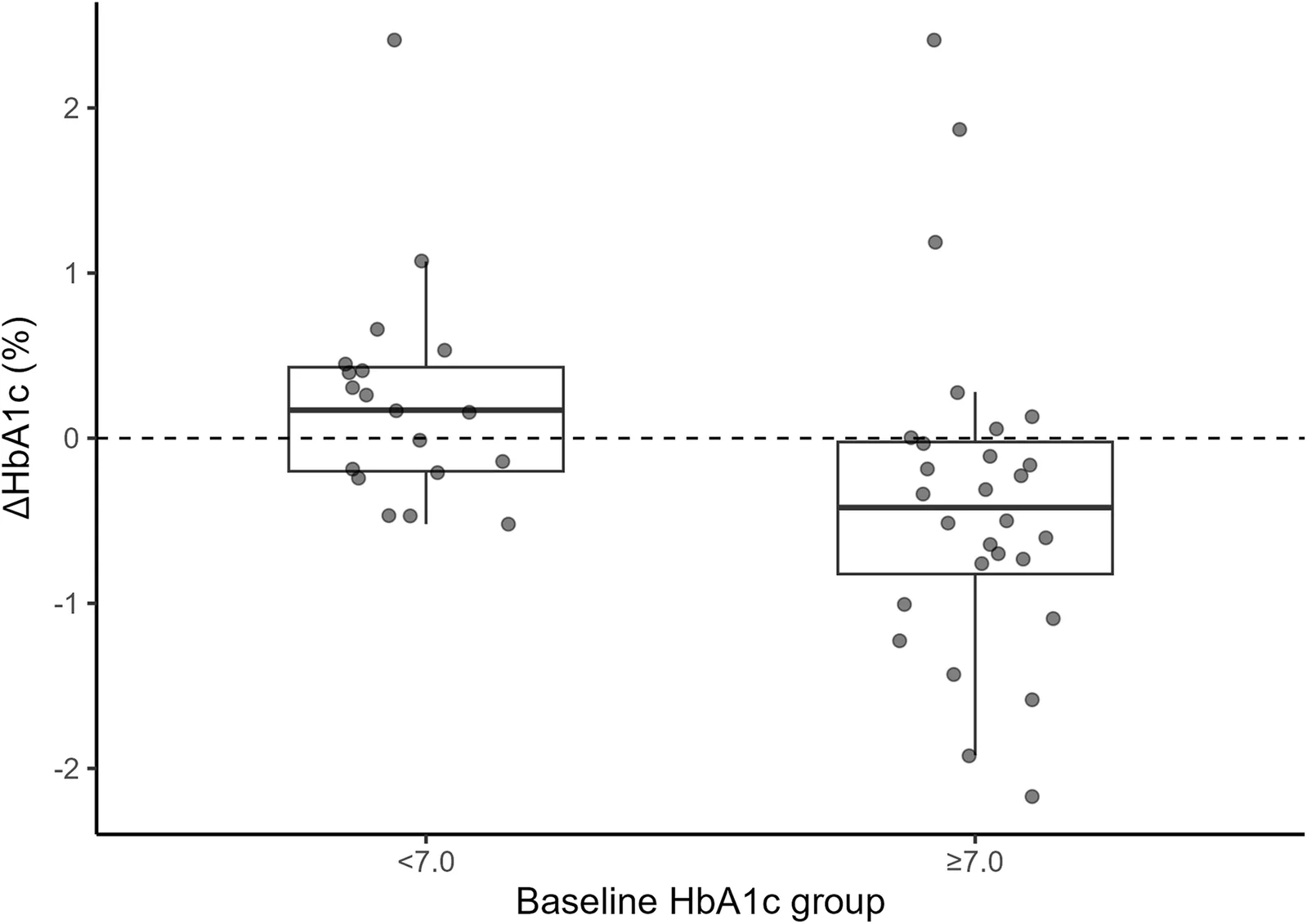
Distribution of changes in HbA1c after digital personalized diet therapy stratified by baseline glycemic control. Boxplots represent the median and interquartile range, and dots indicate individual patients. The dashed line denotes no change from baseline.

Figure 2 summarizes the median changes in energy intake, BMI, and HbA1c following digital personalized diet therapy.

**Figure 2:**
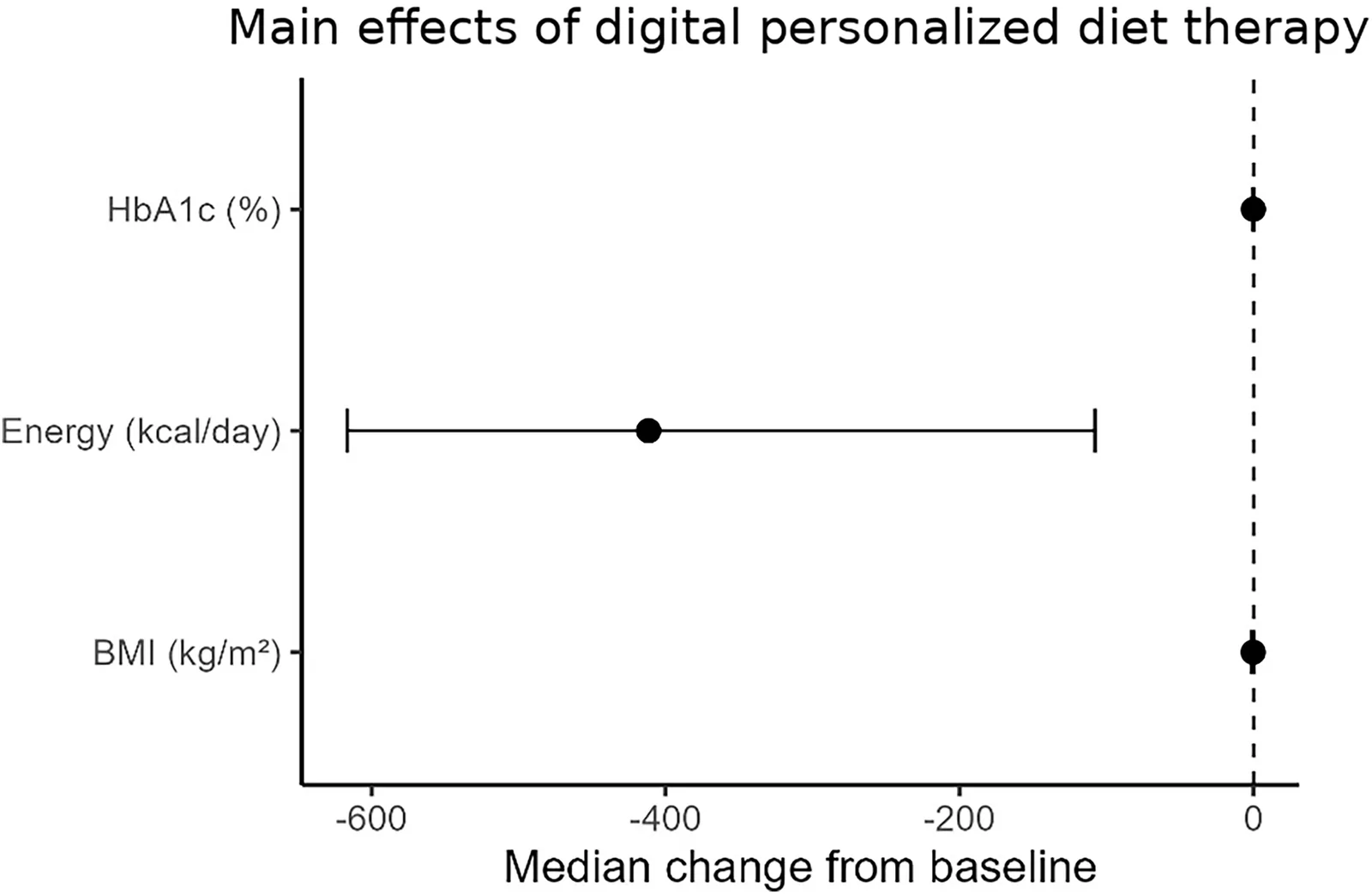
Median changes in total energy intake, BMI, and HbA1c after digital personalized diet therapy. Error bars represent interquartile ranges.

## Discussion

The present real-world study demonstrated that the effectiveness of digital personalized nutrition in type 2 diabetes was not uniform across patients, but instead it appeared to vary according to the baseline glycemic status. Thus, instead of producing a consistent population-level reduction in HbA1c, the intervention resulted in a non-uniform response pattern, with clinically meaningful improvement observed primarily in individuals with elevated baseline HbA1c. These findings should be considered as hypothesis generating, and they highlight the heterogeneous response rather than a uniform treatment effect.

These findings suggest that current digital dietary approaches may not produce uniform benefits across all patients because of substantial inter-individual variability in metabolic response. In this context, digital nutrition may function less as a generalized glucose-lowering strategy and more as a targeted corrective tool for patients with suboptimal glycemic control.

Previous studies of digital interventions for diabetes have consistently reported statistically significant but clinically modest reductions in HbA1c^6^^,^^9^^,^^14^^,^^15^, suggesting that access to digital support alone may be insufficient to achieve meaningful metabolic improvement in unselected patient populations. This is consistent with a recent systematic review and network meta-analysis that reported a pooled reduction in HbA1c of approximately 0.37% across digital health interventions, where the effects diminished over longer follow-up periods,^16^ further supporting the notion that population-level effects may mask clinically important subgroup-level heterogeneity.

Overall, a clinically meaningful glycemic response (HbA1c reduction ≥0.5%) was achieved by 15/47 patients (31.9%), including 14/28 (50.0%) of those with baseline HbA1c ≥ 7.0% versus 1/19 (5.3%) of those with baseline HbA1c < 7.0%, further illustrating the different treatment responses according to baseline glycemic status. According to a multivariable linear regression model of the magnitude of HbA1c reduction on baseline HbA1c, age, sex, BMI, diabetes duration, and baseline energy intake, baseline HbA1c remained significantly associated with the degree of glycemic response after adjustment (β = 0.205, 95% confidence interval (CI): 0.041–0.369, *p* = 0.019), whereas none of the other covariates were significant. The overall explanatory power of the model was modest (R^2^ = 0.164, adjusted R^2^ = 0.039), reflecting the limited sample size relative to the number of covariates, and these findings should be interpreted as exploratory rather than confirmatory. The standardized coefficient for baseline HbA1c was β′ = 0.37, indicating a moderate effect in relative terms. Variance inflation factors were below 1.3 for all covariates, indicating no meaningful multicollinearity. However, the residuals departed from normality (Shapiro–Wilk *p* < 0.001), and non-parametric bootstrap (5000 resamples) yielded a wider 95% CI for the baseline HbA1c coefficient that crossed zero (−0.043 to 0.389) compared with the parametric estimate. This indicates that the regression findings were not robust to distributional assumptions, and thus should be interpreted as exploratory rather than confirmatory, and not treated as evidence that baseline HbA1c is an independent predictor of treatment response.

The intervention produced measurable dietary and anthropometric changes despite no changes in pharmacotherapy, supporting a behavioral mechanism of action under routine care conditions. In particular, changes in HbA1c were not significantly correlated with reductions in energy intake or BMI, suggesting that glycemic improvement was not driven only by caloric restriction or weight loss but may have reflected individualized metabolic or behavioral responses facilitated by personalized dietary guidance.^1^^,^^12^^,^^17^

Importantly, the absence of a significant overall reduction in HbA1c at the population level appeared to reflect response heterogeneity rather than any lack of treatment effect. Patients with baseline HbA1c ≥ 7.0% exhibited a consistent and clinically meaningful decline in HbA1c, whereas changes were minimal in those with well-controlled baseline glycemia. This pattern aligns with the concept of diminishing marginal glycemic benefit near target HbA1c levels and suggests that baseline metabolic status may be associated with differences in treatment responses.^3^^,^^13^

The individual-level HbA1c trajectories also support this interpretation, with a clear downward trend in patients with elevated baseline HbA1c and a stable pattern among those with adequate baseline control (Figure 1). Together, these findings suggest that baseline glycemic status was associated with differences in treatment response beyond what could be explained by statistical artifacts alone.

The intervention was associated with consistent changes in dietary intake, including reductions in major macronutrients and modifications in dietary fat composition, indicating meaningful behavioral adaptation in routine care conditions. The observed decrease in absolute dietary fiber intake could be attributed to the overall reduction in total energy intake rather than a deterioration in diet quality, reflecting a global reduction in food consumption rather than selective nutrient imbalance (Figure 2).

Importantly, the maintenance of unchanged antidiabetic pharmacotherapy throughout the observation period supports the attribution of observed metabolic changes to the dietary intervention itself. In addition, background pharmacotherapy may have attenuated the magnitude of glycemic improvement in patients with already controlled baseline HbA1c, potentially contributing to the absence of a significant overall population-level effect.

Controlled dietary intervention trials have demonstrated that structured nutritional strategies can improve glycemic control under experimental conditions,^13^^,^^18^ but their effective translation into routine clinical practice remains inconsistent, particularly in low-intensity or self-directed settings. In this context, the real-world findings obtained in the present study provide insights into the variable response under everyday care conditions and support a more individualized, stratified approach to the implementation of digital dietary interventions.^7^^,^^10^^,^^19^

The real-world design is a major strength of this study because it reflects routine clinical practice rather than controlled behavioral settings, and thus is directly applicable to primary care environments where intensive nutritional supervision is not feasible.

Several limitations should also be considered. The study employed a pre–post design without a parallel control group, limiting causal inference. However, the consistency of the subgroup-specific effects and stability of pharmacotherapy throughout the observation period support the robustness of the observed associations. The sample size was moderate and the follow-up period was relatively short, limiting the assessment of long-term sustainability. Dietary intake was self-reported, and thus subject to reporting bias, and the participants comprised a motivated outpatient population, which may restrict generalizability to less engaged groups.

Regression to the mean cannot be entirely excluded, but the differential response pattern according to baseline HbA1c and the absence of improvement in patients with well-controlled glycemia do not support this explanation as the only driver of the observed effects. Given the number of statistical comparisons performed across subgroups, dietary variables, and correlation analyses, the possibility of a type I error cannot be excluded. The pre-specified nature of the primary subgroup comparison (baseline HbA1c < 7.0% vs ≥ 7.0%) mitigates but does not eliminate this concern, and formal correction for multiple comparisons was not applied.

From a primary care perspective, these findings are particularly relevant because digital dietary interventions are increasingly implemented in routine outpatient settings with limited resources and without intensive supervision. Identifying patients who are most likely to benefit, such as those with elevated baseline HbA1c, may improve the efficiency of these interventions and support more targeted clinical decision making. From a clinical standpoint, the findings suggest that digital personalized diet therapy may be most beneficial for patients with suboptimal baseline glycemic control, supporting a targeted rather than universal implementation strategy. Given the observational design, these findings should be interpreted as associations rather than causal effects.

## Conclusions

Digital personalized diet therapy in routine clinical practice resulted in a variable glycemic effect rather than a uniform population-level response. Baseline HbA1c was associated with the glycemic response to digital personalized nutrition and may help identify patients who are more likely to benefit from this intervention.

These findings support a more targeted approach to the implementation of digital dietary interventions by prioritizing patients who are most likely to benefit. However, given the observational design of this study, these findings should be interpreted as associations and require confirmation in larger prospective studies with appropriate control groups.

## Informed consent

Written informed consent was obtained from all participants prior to inclusion in the study.

## Authors’ contributions statement

All authors testify that all persons designated as authors qualify for authorship and have checked the manuscript for plagiarism. If plagiarism is detected, all authors will be held equally responsible and will bear any sanctions imposed by the journal.

All authors meet the following criteria: (1) substantial contributions to the conception and design of the work, or acquisition, analysis, or interpretation of data; (2) drafting the work or revising it critically for important intellectual content; (3) final approval of the version to be published; and (4) agreement to be accountable for all aspects of the work.

## Ethical approval

The study was conducted in accordance with the Declaration of Helsinki and approved by the Local Bioethics Committee of West Kazakhstan Marat Ospanov Medical University (Protocol No. 16-2024).

## Data availability

The data sets generated and/or analyzed during the current study are available from the corresponding author on reasonable request.

## Author contributions (CRediT statement)

Indira Kaibagarova: Conceptualization, Methodology, Investigation, Data curation, Formal analysis, Writing – original draft, Writing – review & editing, Visualization.

Aigul Sartayeva: Supervision, Validation, Writing – review & editing.

Nurgul Ablakimova: Data curation, Investigation, Writing – review & editing.

Gaziza Smagulova: Investigation, Data curation.

Alla Ovsiannikova: Validation, Supervision, Writing – review & editing.

All authors have read and approved the final version of the manuscript.

## AI statement

AI-assisted tools were used to improve language and readability. All content was critically reviewed and approved by the authors, who take full responsibility for the final manuscript.

## Funding

This research received no external funding.

## Conflict of interest

The authors have no conflicts of interest to declare.
