# Supplementary material for: Baseline HbA1c is associated with glycemic response to digital personalized nutrition in type 2 diabetes: A real-world study
Source: J Taibah Univ Med Sci. 2026 Sep 22;21(5):1039–46. doi: 10.1016/j.jtumed.2026.09.006 (PMC13654518; doi:10.1016/j.jtumed.2026.09.006)
Supplement: Supplementary file 1 [file mmc1.docx]

*Figure S1 (STROBE flow diagram): Participant flow through enrollment, follow-up, and final analysis.*
